# The Lived Experience of Critically-Ill Muslim Patients in Isolation

**DOI:** 10.1080/17482631.2022.2032548

**Published:** 2022-02-08

**Authors:** Sondos B. Eqylan, Reema R Safadi, Valerie Swigart

**Affiliations:** a Clinical Nurse Specialist, Al-Hussein Salt New Hospital, Salt, Jordan; b Maternity and Child Health Nursing Department, School of Nursing, The University of Jordan, Amman, Jordan; c Professor, School of Nursing, University of Pittsburgh, Pittsburgh, Pennsylvania, USA

**Keywords:** Critically-ill patients, isolation experience, infectious disease, Muslim, isolation precautions, phenomenology

## Abstract

**Purpose:**

This study explored critically ill Muslim patients’ experiences and perceptions related to confinement to isolation rooms.

**Methods:**

The descriptive–interpretive lens of phenomenology was employed to explore and illuminate the isolation experience of critically ill Muslim patients). Semi-structured, face-to-face, audiotaped interviews were conducted . Colaizzi’s method of data analysis, in combination with an interpretive analysis supported by van Manen’s “lifeworld constituents” were used.

**Results:**

Data analysis revealed four themes: Feeling isolated and imprisoned; losing basic patients’ rights; feeling rejected by healthcare providers; and accepting isolation and its adversity. Findings were illuminated by applying van Manen’s lifeworld constituents: spatiality, temporality, relationality and corporeality. The patients described the overwhelming impact of isolation on their physical, emotional, social and spiritual health.

**Conclusions:**

This study provides healthcare providers with an in-depth understanding of critically ill patients’ physical, psychological and spiritual needs. Although the unique needs of Muslim patients are highlighted, it is evident that patients’ suffering in isolation is universal. Healthcare providers are encouraged to consider creative measures to support and help patients cope with the adversity of isolation.

## Introduction

Infections and the required protective isolation procedures are a great burden to health care services and healthcare professionals. Sepsis related to acquired infection is one of the major leading causes of morbidity and mortality in intensive care units (ICUs), and it is the most common cause of critically ill patients’ admissions and readmissions to hospitals (Genga & Russell, 2017). critically ill patients have an increased risk of developing infections either because of having a critical illness and compromised immune systems, or due to acquiring healthcare-associated infections while hospitalized (Centers for Disease Control and Prevention [CDC], 2017; Magill et al., 2014). The Centers for Disease Control and Prevention (CDC) recommends applying isolation regulations as soon as an infectious disease is suspected. Isolation protocols are considered a mandatory standard practice to provide safety to patients and staff.

The isolation experience is worsened when combined with a critical illness (Evans et al., 2003). In the ICU, a critically ill patient with infectious disease is subject to an unpleasant environment permeated with noise, ambient light, alarms, electronic devices and monitors, and above all, by mandatory isolation restrictions. This stressful environment is magnified when critically ill patients must endure more restrictions such as strict visiting hours and limited mobility compared to other patients in the general ICU rooms.

A review of the literature revealed numerous studies describing the negative impact of isolation on patients’ physical, psychological and social well-being. Higher incidence of depression and anxiety, increased anger/hostility scores, perceptions of stigma, feelings of fear, loneliness, and mood disturbances have been reported (Abad et al., 2010; Barratt et al., 2010; Gould et al., 2018; Guilley et al., 2017). In addition to the emotional and psychological impact of isolation, studies have found that patients requiring contact isolation precautions were eight times more likely to fall out of bed, develop pressure injuries, and suffer fluid and electrolyte imbalance when compared to patients not under isolation precautions (Gould et al., 2018; Stelfox et al., 2003). Researchers also found that healthcare providers spend less time in providing direct patient care to patients in isolation than with patients in non-isolation areas; this led to lower satisfaction rates with care (Dashiell-Earp et al., 2014; Shaban et al., 2020).

Although this study was conducted before the COVID-19 pandemic, the results of this research have become more relevant as overwhelming numbers of patients have required ICU care with strict isolation procedures, and healthcare workers struggle to care for these patients while protecting themselves and their families. Recent studies have described the extreme strain on healthcare systems, healthcare providers and patients (Chaccour, 2020; Sahoo et al., 2020). However, a search of the major databases (PubMed, Sage, CINAHL, and EBSCO host) revealed no qualitative studies focusing on the isolation experience of critically ill Arab-Muslim patients. This study addresses this gap in knowledge using phenomenological inquiry. Phenomenology is used to give voice to Muslim patients who suffer the compound effect of critical illness and isolation. Therefore, this study aims to explore critically ill Muslim patients’ experiences and perceptions related to confinement to isolation rooms in Jordan.

## Methods

### Design

The descriptive-interpretive lens of phenomenology, described by Max Van Manen (1997) and the procedural steps suggested by Colaizzi (1978) were used to explore and illuminate the isolation experience of critically ill Muslim patients.

### Study setting

This study was conducted in the ICU of a private hospital in Amman, Jordan. The isolation rooms are walled-off sections on this unit. The rooms have a single bed, with an area that ranges between 12.25 and 14 square metres. A single-sided signpost indicating “Isolation Room” and the type of isolation precautions is hung on the door of patient’s room. This signpost is visible to those outside the room, i.e., healthcare providers, visitors, and other patients, but not to the patient inside the room. critically ill patients in isolation settings are connected to a cardiac monitor and multiple invasive or non-invasive machines. Healthcare providers are instructed to take standard precautions and transmission-based precautions (either contact, droplet, airborne or these in combination) when they provide direct patient care.

### Ethical approval processes

The Institutional Review Board at the University of Jordan, the Research Deanship, and the School of Nursing approved this study. Ethical approval was obtained according to the University of Jordan graduate studies policies and from hospital ethics committee. The researcher followed ethical guidelines to protect participants’ rights including the right to voluntary participation, privacy, and confidentiality. Voluntary participation was facilitated by the ICU nurses who were offered information about the purposes and procedures of this study and were invited to help recruit eligible patients. The nurses approached patients who were being discharged from the ICU and offered them a cover letter and information sheet. Patients were invited to contact the research by phone once they were settled on a regular ward. Informed consent was obtained and face-to-face interviews were conducted when the patient’s condition was stable in a private area on the hospital ward.

### Participants

critically ill patients who had been confined to ICU isolation rooms were recruited using purposive sampling technique. Inclusion criteria included being a critically ill patient, over the age of 18 years old, and diagnosed with a suspected or actual infectious disease requiring isolation for a minimum of 72 hours. A primary condition was that patients were not intubated or sedated during their stay in the isolation room and had a Glasgow Coma Score of 15/15.

### Demographic data

Ten patients (5 males and 5 females) participated in face-to-face interviews after discharge from the isolation settings in ICU. Patients’ characteristics (age, educational level, marital status, employment, infection status, duration and type of isolation, and medical diagnosis on admission) are illustrated in Table I.

**Table I.** Characteristics of participants *(N = 10).*

Participants’ Characteristics	n (%) or M± SD
**Age**	42.2 **± **17.3
**Sex**	
Female	5 (50)
Male	5 (50)
**Educational level**	
High school	2 (20)
College/ University	8 (80)
**Marital status**	
Married	6 (60)
Unmarried	4 (40)
**Employment status**	
Yes	6 (60)
No	4 (40)
**Infection status** Confirmed	8 (80)
Suspected	2 (20)
**Duration of isolation stay**	6.6 **± **2.2 days
**Type of isolation precautions**	
Contact precautions	5 (50)
Droplet precautions	4 (40)
Airborne precautions	1 (10)
**Medical diagnosis on admission**	
Cardiopulmonary diseases	6 (60)
INFECTIOUS diseases	2 (20)
Other	2 (20)

### Data collection

This study was conducted over 3 months in 2019. Face-to-face audio-recorded interviews were conducted in a quiet and private place at the patient’s convenience, and within 24 hours of transfer from the isolation room in the ICU to the regular ward. At the onset of an encounter with a potential participant, the researcher explained the study purpose and procedures and invited each participant to sign an informed consent for participation. Permission to tape-record the interview was obtained before beginning the interview. Interviews were conducted in Arabic language. The interview started with introductory and demographic questions. This was followed with in-depth, semi-structured questions using an interview guide designed for the purpose of this study.

An interview guide with open-ended questions was developed based on the literature (deMarrais, 2004; Rubin & Rubin, 2012). The guide used Patton’s six foci: experience/ behaviour, opinions/ values, feelings, knowledge, sensory impressions and demographic data (Patton, 2014; Roulston, 2014). Examples of the questions asked were: (1) Describe your experience while you were in the isolation room. (2) How did you feel when you were in isolation? (3) From your own perspective, what factors influenced your satisfaction/ non-satisfaction with the isolation experience. Additional probing questions were used as required to elicit further details of an account. Examples of these expressions are: “Tell me more about how you felt then” or “Walk me through that incident as it occurred.” The interviews lasted 25–40 minutes. In this study, sampling continued until the researchers determined that no new information was emerging during interviews and data saturation was reached.

### Data analysis

According to Van Manen (1997) the researcher is engaged in an existential investigation, exploring the phenomenon in generating data and using personal experience as a starting point. After several readings and re-readings of the transcripts, independent line-by-line coding by the researcher and the advisor were compared and agreed upon by reaching a consensus on the significant statements and their codes. The coded statements were translated by the researchers and a copy of the Arabic and English translation was reviewed by a professional bilingual translator. The translator compared both versions and ascertained that the Arabic original meaning was preserved.

Seventy-one formulated meanings were derived from the significant statements (Table II). Following agreement on all formulated meanings, the meanings were organized into 10 sub-themes. The emergent sub-themes were then condensed into four main themes and then validated in the context of the original statements of the patients. The researchers then compared their clusters of themes and checked the accuracy of the overall thematic map. The results were integrated into in-depth descriptions of the phenomenon under study.

**Table II.** Examples of the process of creating formulated meanings from significant statements

Significant statements	Formulated meanings
*“My experience in isolation room was like I was locked up in a prison, in solitary confinement, all alone.”* (Transcript 1, page 1, lines 1–3)	Feeling of imprisonment in a narrow and small place.
“*In isolation room, a day would feel like two days, and a one hour like five hours, time passed slowly*.” (Transcript 2, page 2, lines 20–21)	Losing sense of time (a long day, time is slow)
“*Suddenly while I was in the intensive care unit they [healthcare providers] came for my room, closed its door, and they hung an isolation sign on the glass door, but I did not realize why did they do that or what was wrong with me. They did that without providing me any explanation.”* (Transcript 7, page 2, lines 16–20)	Shunned from knowledge about one’s own health condition
“*From that day, their behaviors and attitudes changed, they stopped coming in to my room, they stopped talking to me, they would come and do their job as if I was not there, and then they would just leave*.” (Transcript 10, page 3, lines 35–39)	Nurses’ behaviours changed
“*nurses were more concerned with monitoring devices and machines rather than asking me about my needs and feelings. They never showed interest in understanding how I felt or what I needed.”* (Transcript 9, page 3, lines 15–19)	Ignoring psychological needs
“I did not even have my eyeglasses. Swear to God, I was unable to properly see the faces of the people I talked to, not even see what I was eating.” (Transcript 10, page 4, lines 46–51)	Separated from personal belongings

At completion of analysis, we used the work of Van Manen (1997) to further illuminate how individuals’ realities are influenced by restrictions of isolation. We employed van Manen’s four constituents of one’s lifeworld: spatiality or lived space, temporality or lived time, relationality or lived human relations, and corporality or lived body to provide a framework for illumination of the isolation experience.

Trustworthiness of the study

Beck (1994) noted that using criteria of truth value, applicability, consistency, and neutrality is appropriate for phenomenology research. Truth value was determined by keeping field notes and verbatim transcripts across all stages of the study. Applicability was achieved by obtaining in-depth descriptions from the participants. Consistency was achieved by the researchers’ collaboration through the processes of data coding, categorizing, translating, and creating themes that reflected patterns throughout the data. Neutrality, which refers to freedom from researchers’ bias, was achieved through the researchers’ openness, respect, and non-judgemental attitude in data collection and analysis.

## Findings

critically ill Muslim patients’ experiences confinement in isolation rooms resulted in four themes: (1) Feeling isolated and imprisoned, (2) Losing basic patients’ rights, (3) Feeling rejected by healthcare providers, and (4) Accepting isolation and its adversity. Table III displays the emerging sub-themes that were grouped into main themes.

**Table III.** Constructed themes and subthemes

Main themes	Sub-themes
Feeling isolated and imprisoned	1- Being trapped in space and time 2- Suffering emotionally
Losing basic patient’s right	1- Right to be informed 2- Right to preserve belongings 3- Right to have adequate care 4- Right to be respected and have dignity
Feeling rejected by healthcare providers	1- Feeling Avoided by healthcare providers 2-Impaired communication (reaction and interaction) with healthcare providers
Accepting isolation and its adversity	1- Being considerate of hospital rules and regulations 2- Being considerate of staff’s roles and responsibilities

### Theme 1- feeling isolated and imprisoned

critically ill patients in this study often discussed their personal concerns about the structure of the isolation rooms, the hospital’s rules and regulations requiring being confined to isolation, and the time spent in the isolation setting. For these patients, the small and narrow rooms felt like a prison. This sense was conveyed by a 21- year-old female patient (5), a suspected case of tuberculosis under airborne isolation precautions, “*My experience in isolation room was like I was locked up in a prison, in a solitary confinement, all alone*.” In addition, patients expressed feelings of being isolated and distant from their families and the external world. Discontent with separation from loved ones was expressed by a 26-year-old male patient (4), who was under contact isolation precautions, said: *“My family and loved ones were not by my side, they never allowed them to visit me, and I was separated from them when I needed them the most,”*

Furthermore, patients described intensive emotional expression of loneliness, stress, boredom and fear because of being separated from the external world. One patient expressed her loneliness by saying: “*loneliness is a scary feeling; it is scary when you are separated from the people who are supposed to be with you in time of stress, to help you get through difficulty*.” Another patient (7), 42 years-old, who was diagnosed with asthmatic attack, under droplet isolation precautions, expressed his feelings of stress in the following words: *“I can just say that this is by far the hardest and worst experience anyone can go through. I felt stressed most times.”* Boredom was a prominent feeling. Several patients described their feelings about time passing that was perceived as long and boring. One patient said, “*I used to feel like time was stagnant. I felt it was much longer; one day in isolation was like three days*.” Patients’ feelings of fear were varied between fear of dying alone and fear of the unpredictable news. Patient (6), who was under contact isolation precautions, expressed her fear by saying: *“I was scared that I would die with no one holding my hands.” “*Unpredictable news” and “not knowing what would happen” were scary feelings too. A 26-year-old female participant (10), who was diagnosed with meningitis, commented, *“ …, it was like there is something they just do not want me to know about, I was scared*.”

### Theme 2- losing basic patients’ rights

The patients complained about not being treated the way patients should be treated. All patients criticized being deprived of their rights (1) to get information about their own health condition, (2) to keep their personal items and security belongings (eye glasses or religious items such as rosary and the Holy Qur’an), (3) to have adequate physical, psychological and spiritual care, and (4) to be respected and have dignity.

Patients reported that they received minimal information regarding their isolation condition. They asked for their rights to be informed about their illness and health condition, and to not have to deal with unexpected or hidden news. This concern was experienced by patient (8), who was under droplet isolation precautions because of Novel H1N1 associated pneumonia: *“No one ever explained to me why I was admitted to that room! I did not know why, nor what was wrong with me. Two days later, the doctor finally told me why!”*

Separation from personal and security belongings was another concern. Patient (10) described yearning for her personal belongings by saying:

*All my personal belongings and possessions were taken away from me: My phone, my slipper, my dentures, and even my glasses. I didn’t know why; these are my personal things. The nurses and doctors in isolation rooms assumed that we should be detached from the external world!!*

Deprivation of physical, psychological, and spiritual rituals was the third complaint of patients confined to isolation. Several patients described a change of care as they were diagnosed with an infectious disease. Participant (4) explained that experience succinctly: *“Because of this germ, nurses did not even change my position as frequently as they did before I was diagnosed with this infectious disease. My lower back sore worsened day after day!!”*

Additionally, patients complained about their psychological needs being ignored. From the patients’ perspective, ignoring psychological needs by healthcare providers was only one facet of not receiving adequate care while staying in isolation rooms. A 49-year-old male patient (9), who was under contact isolation precautions, described such an experience in the following words: *“nurses were more concerned with monitoring devices and machines rather than asking me about my needs and feeling. They never showed interest in understanding how I felt or what I needed.”* Moreover, three of the patients expressed their dismay for ignoring their spiritual needs. One patient said *“I could not read the Holy Qur’an in that room; it was not available and I could not get mine into the room. I asked the nurse twice to bring me one, but she ignored my request.”*

The fourth and the last of the reported deprived rights was patients’ loss of their rights to respect and dignity. Several patients described how isolation had affected their sense of dignity. Patients sensed a feeling of disrespect that was related to healthcare providers’ stigmatization of the infectious disease. Patients felt unwanted and dehumanized. Patient (7) expressed feeling stigmatized in the following words: *“mmm, every time there was a reason for them to walk into my room they would loudly exchange this conversation: look out and be careful; she’s an isolated patient, an isolated patient, I really felt I had scabies.”*

Furthermore, dehumanization was another complaint that signalled disrespect of one’s dignity. This was stated by a female patient (2), who was under contact isolation precautions and felt displayed like animals in a zoo*: “I was like a captive animal who was locked away in a cage in the middle of a zoo, everyone outside was staring at me from behind the barred doors and windows.”*

### Theme 3- feeling rejected by healthcare providers

At the beginning of the interviews some patients were reluctant to tape-record the interview. Some others changed the topic when a healthcare providers showed up unexpectedly. After building a good rapport with them, they confided to the researcher that they wanted their stories hidden from healthcare providers. One participant stated, “*If nurses and doctors know about what I just told you, I am afraid they will be totally annoyed with me and withhold their care, so please keep my story between the two of us.”*

critically ill patients criticized healthcare providers’ caregiving, and they complained about their competencies and the way they managed their care in isolation settings. Experiences of being rejected and avoided by healthcare providers were reported by one patient (10) who explained, “*I would see them outside my room arguing about whose turn it was to come into my room and give me care. NO one really wanted to be my nurse on that shift.”* Similar perceptions were echoed by patient (4) who said: *“Once I became really ill and my condition worsened, the doctor did not even come in to check on me until it was too late, all because of the contagious pathogen I had.”*

Moreover, they complained about the quality of communication and interaction with healthcare providers, and reported that healthcare providers spent less time with them than what they had expected in the isolation room. Patient (1), who was under droplet isolation precautions due to an infectious plural effusion, eloquently described her suffering: *“Doctors and nurses would come into my room, do what they should do, and leave as fast as lightning ‘rocket speed’.”*

In addition to avoidance and rejection of patients in isolation, the patients believed that healthcare providers were extra cautious in protecting their “own selves” using exaggerated precautions of distancing, and avoiding contact and communication instead of reassuring patients by explaining the reality of the illness. Patient (4) narrative expressed this fear: *“Doctors and nurses talked to me while keeping their distance; my hearing was not good, and their low voices made the communication strenuous.”* Furthermore, a similar sentiment was echoed by patient (3) in sharing her experience of interaction with healthcare providers, when she was isolated under contact precautions, in the following words: *“What upsets me most was that nurses hated coming into my room or talking to me. It was as if this germ was transmitted by air or by talk, who knows?”*

### Theme 4- accepting isolation and its adversity

All patients in this study concluded their accounts by accepting isolation confinement and its adversity. They expressed respect for the staff and hospital rules and regulations. Patient (5) described her concession with hospital’s rules regarding family visiting in the following words: *“I am not upset with nurses preventing my family from visiting me, I am scared that they might get infected. I don’t want them hurt.”* Furthermore, the patients tried to understand the healthcare providers’ position, and justified their behaviours of rejection and impaired communication as due to fear of getting infected themselves. One patient stated, “*Nurses and doctors were definitely scared that they would get the infection. They would think that way, and it is their right to be distant from me.”*

## Discussion

**Figure 1.**
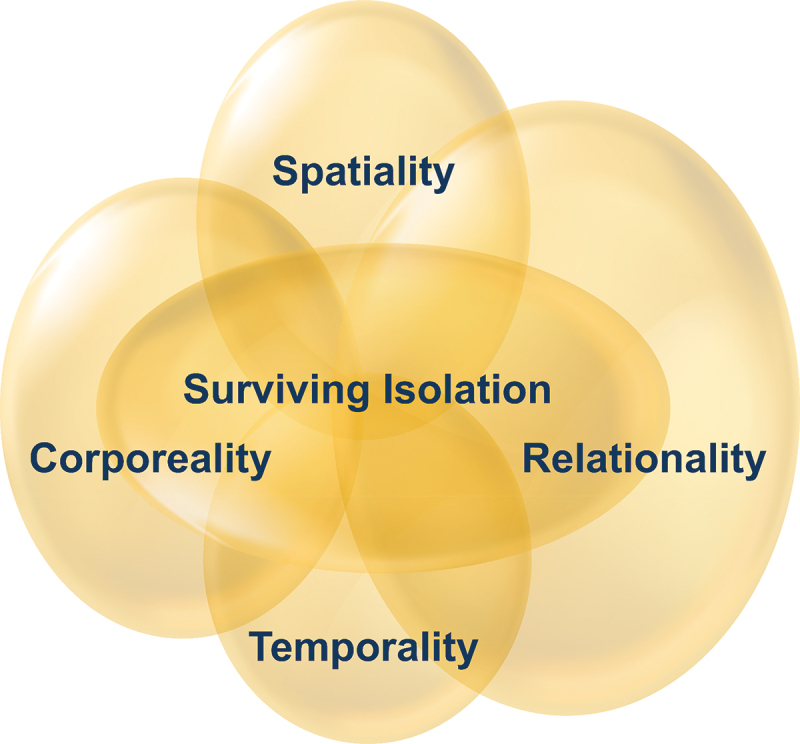
Four lifeworld constituents (van Manen, 1997).

The findings of this study reveal that critically ill patients feel lonely, imprisoned, rejected, deprived of their rights, and dissatisfied with healthcare providers’ care provision during their confinement to isolation rooms. Yet, they accepted the restrictions and endured the adversity. In the phenomenological literature, researchers often employ four lifeworld constituents as a framework for discussion of findings (Eggenberger & Nelms, 2006; Garrett, 2010). The lifeworld constituents, first defined by Van Manen (1997), are: the lived space or spatiality, lived time or temporality, lived human relations or relationality, and the lived body or corporality. The lifeworld constituents will be used here to further illuminate how individuals’ realities are influenced by restrictions of isolation (Figure 1). In this sense, we will use van Manen’s four constituents as an extension of the constructed themes in our findings to illuminate the phenomenon under investigation.

### Spatiality

The spatiality dimension refers to the lived space in which individuals are situated, and it reflects the ways that individuals experience day-to-day existence in the world (Munhall, 2012). Patients’ lifeworlds in isolation settings existed within the limited area of a small room. They are confined to that room, separated from their loved ones and their own belongings. This sense of imprisonment within the lived space of isolation disrupted the individuals’ normal life and suspended their daily routines. They experienced both sociological and psychological impacts, feeling entrapped in space, time, and absent of the comfort of family and loved ones. These conditions created feelings of loneliness and consequently fear of dying alone. These findings are consistent with previous literature results that revealed that the physical context and structural design of isolation settings have a negative impact on patients’ mood and increases rates of fear, boredom and loneliness, (Barratt et al., 2010; Biagioli et al., 2016; Skyman et al., 2010).

### Temporality

The phenomenological perspective of time is captured in a remark attributed to Einstein that expresses the difference between embodied time and chronologic time: “Put your hand on a hot stove for a minute and it seems like an hour. Sit with a pretty girl for an hour and it seems like a minute. That’s relativity.” (Starks & Trinidad, 2008). The perception of time changes with our life situation; Brown et al. (2006) noted that Time was described as a constant consideration for patients waiting for liver transplant as moving very fast and stretching out all at once, and slows down as they wait for the transplant (Brown et al., 2006). In this study, the inner time consciousness among critically ill patients in isolation settings differed from external real time; they had different perceptions of time passing due to the lack of options they had for spending that time. Consequently, a “new” meaning of time or perception of time was prominent and was described as lingering and meaningless. These findings are consistent with several studies that found that strict rules and regulations for separating and isolating patients from the external world engendered feelings of boredom (Biagioli et al., 2016; Shaban et al., 2020; Skyman et al., 2010).

### Relationality

The relationality concept refers to “the lived human relations we maintain with others in the interpersonal space” (Van Manen, 1997). Confinement to an isolation environment limits and alters patients’ social relationships and interactions with their families and loved ones while creating new stressful relationships and interactions with healthcare providers. These findings are congruent with Barret and colleagues’ results, which showed that the restrictions in isolation settings reduced patients’ opportunities for socializing and interacting with others (Barratt et al., 2010). In this study, patients in isolation settings clearly described their unmet needs and expectations. They identified three areas wherein healthcare providers failed to relate or act effectively: inadequate provision of information, inadequacy of physical care, and impaired interactions revealing lack of respect. Inadequacy of provision of information created a loss of trust in the source of information, escalation of fears and anxiety as they imagined all sorts of unpredictable news. These findings are consistent with the study of Guilley et al. (2017) which revealed that 67% of the patients under isolation precautions in Western France were not satisfied with the quality of information they received and this increased their anxiety (Guilley et al., 2017).

The second relationality concern among the patients in this study was about quality of physical care received. They described care received as poor, incompetent, and less than the optimal care they should have received. These findings are consistent with the literature about patients in isolation settings described as having fewer vital signs recordings, more days without physicians’ notes in their charts, higher risks of falling, pressure injuries, and fluid and electrolyte imbalance compared with patients who were not placed in isolation settings (Gould et al., 2018; Stelfox et al., 2003).

The third concern of patients in this study was centred around impaired interaction with healthcare providers during their confinement to isolation rooms. Culture is significant in shaping an individual’s perceptions about health and his/her relationship with healthcare providers (Betancourt et al., 2003). The patient-healthcare provider relationship is threatened when the cultural meaning of this relationship is violated during hospitalization (Johnstone & Kanitsaki, 2006). Patients in this study reported minimal interactions with healthcare providers and hurried medical procedures. Previous studies found that isolation adversely affects patient-healthcare providers’ communication and relationships (Barratt et al., 2010; Shaban et al., 2020; Skyman et al., 2010). Healthcare providers perceive patients in isolation settings as different from other patients and admitted that they spend less time in direct patient care (Cassidy, 2006; Gill, Kumar, Todd, Wiskin, 2006).

### Relationality and islamic culture

In this study, van Manen’s concept of relationality was used to interpret patients’ descriptions of impact of isolation on their relationships with family and religion (spirituality or belief in a greater power or God). It is in this area that the patients, all members of the Islamic culture, reported the most anguish. The people of Jordan are a collectivist society having two essential elements in their lives: religion and family. In the isolation setting, these patients repeatedly expressed the acuteness of the loss of both elements; they were painfully anxious about losing contact with their family and distraught at losing all material elements necessary for their practices of spirituality.

In Islamic culture, visiting a patient has a religious value; family members are passionate towards the ill person and want to be close to their significant other during critical illness. Illness is a time to express their love, closeness, and empathy. These feelings were clearly explained by Othman et al. (2020) and Imam Nawawi (1987) who elucidated the deeply felt obligation of Muslim families to be beside their patient/family member in critical situations. In this study, although patients were stressed because of the separation from their loved ones, they understood the situation and wanted to protect them; this was a kind of consolation, after all, this is part of their commitment and closeness to their family.

Ignoring spiritual needs was less tolerated. Patients in this study were critical of healthcare providers’ seeming to ignore spiritual needs. Muslims are conditioned to ask for Allah’s help in their daily life practices and this becomes more central in critical situations. Rituals such as praying, reading from the Holy Qur’an, and begging Allah for mercy are sources of strength for the Muslim patients and their families. Muslims believe that suffering and enduring illness is a way of testing one’s level of faith and religious commitment; endurance of illness is purifying, and it is rewarded by an everlasting life and access to paradise and God’s blessing (Imam Nawawi, 1987; Othman et al., 2020). Muslims believe that God, in His infinite wisdom, has given them clear guidelines about how to deal with their illness to make it bearable with its afflictions and even to be grateful to Him to pass this examination of endurance. Reading the Holy Qur’an’s words of remembrance and supplication, and a daily five-times prayers are perceived to be the most powerful tranquillity behaviours exercised during this stressful period. Lack of access to the Holy Qur’an interfered with carrying out important spiritual rituals.

### Corporeality

Patients’ consciousness and awareness of being in isolation were formulated through their lived bodies and minds. In phenomenology, the concept of the lived body “refers to the phenomenological fact that we are always bodily in the world” (Van Manen, 1997). In this study, patients’ perceptions of their bodies influenced their actions and thoughts. Patients in isolation described the imposed constraints of movement of their bodies with descriptions such as “being suffocated”, “unable to walk” “restricted to a small room with unopened windows.” Furthermore, in more exaggerated expressions, patients expressed feelings of being stigmatized, dehumanized, unwanted and treated like a captive animal. Patients’ metaphors and imageries were expressive of their imprisoned body within a small space, all imposed upon them because of the pathogen that infected their bodies.

Patients’ bodily perceptions in this study were congruent with other studies’ findings. Studies have reported isolated patients’ descriptions of being vulnerable, stigmatized, having a body that is different from others, “being dirty”, “having the plague”, “having leprosy”, and “being dangerous” (Barratt et al., 2010; Skyman et al., 2016). Furthermore, several studies indicated that although patients have understood healthcare providers’ emphasis on isolation precautions, the exaggerated use increased their sense of being stigmatized (Gammon et al., 2019; Larsen, 2009; Robertson et al., 2004).

### Accepting and surviving

The experience of illness involves not only the physical body, but also affects one’s behaviour, self-image, and relationships (Uchmanowicz et al., 2016). Effective acceptance of a health condition supports positive adaptation to illness. Acceptance also supports the positive relationship between patients having trust in their healthcare providers and the subsequent active role in the treatment plan (Uchmanowicz et al., 2016). In their struggle to accept or cope with the adversity of isolation, patients in this study justified strict rules and regulations and healthcare providers’ distressing interactions as an element reassuring their safety. This finding is congruent with Gasink et al.’s (2008) study that found that 94.9% of the patients who were under contact precautions accepted their isolation status, and felt that the imposed precautions were instituted for their protection and the safety of others. The acceptance of the illness and associated precautions and restrictions can be interpreted as a subconscious resolution that enabled patients to *survive* the severity of the isolation experience with all its challenges. Reaching the stage of full acceptance of the illness was not an easy attainment, and the time required to achieve that probably varies with individual patient’s tolerance, adaptability and resources.

## Conclusions

The current study revealed that in isolation units, critically ill isolated Muslim patients’ experience and needs are universal as well as specific to the Muslim cultural group. This study revealed the centrality of inadequacy of the healthcare system’s and healthcare providers’ attention to the spiritual and religious practices of Muslim patients and their families. The religious practices of Muslims are crucial aspects of their lives; deprivation of these rituals contributes to stress and suffering. Treating patients in isolation as “normal” humans with dignity and rights to practice their spiritual beliefs is a value that applies to all patients regardless of their diagnosis, hospitalization setting, or cultural background.

## Limitations and recommendations

Consistent with the nature of qualitative research this study focused on development of understanding of a specific phenomenon. The focus was one select group of a specific culture within one particular setting. Generalizability was not intended. Limitations were small sample size and the resources of the researcher including time. Studies of the isolation experience of persons with varying age, gender, and culture are recommended.

## Implications

Healthcare providers are urged to consider all aspects of care (physical, psychological, social, cultural and spiritual) when providing care to critically ill patients in isolation. Considering spiritual care is essential in time of stress and it is central for Muslims who rely on their religious beliefs and daily practices for coping. Some suggestions for meeting Muslim isolated patients’ spiritual needs include using disposable (single-use) religious items (prayer rugs, rosary beads, Holy Qur’an stand, prayer clothes, and Islamic prayer clock). A single remotely controlled wall screen in each isolation room that displays verses of the Holy Qura’n digitally could help in attaining comfort while practicing religious rituals and establishing a close relationship with God (Allah).

Due to the necessity for distancing and isolation precautions while keeping empathy and compassionate care, strategies that enhance communication with patients in isolation and their families must be developed. Every effort should be made to allow patients to retain hearing and visual aids. Communication suggestions include using writing boards, voice “messages” messaging such as smartphones or tablets computer devices, video calling applications (e.g., Zoom and Skype).

Enhanced training and surveillance should be provided to personnel engaged in care of critically ill patients in isolation units. Healthcare institutions must establish periodic training programmes for its health staff to maintain an up-to-date information about pathogen transmission and protection measures beside care delivery that covey empathy and compassion to patients and their families.

The findings of this study have universal implications for policy makers, health care institutions’ administrators and care providers administering care to critically ill patients confined to isolation units. The outcome of this study may be very relevant to patients in other Middle Eastern and Muslim countries, and considered timely during the global and recent outbreaks of infectious diseases.
